# Gender differences in the determinants of mature entrepreneurship? The case of Germany

**DOI:** 10.3389/fsoc.2022.998230

**Published:** 2022-12-07

**Authors:** Laura Romeu Gordo, Justyna Stypińska, Annette Franke

**Affiliations:** ^1^Deutsches Zentrum für Altersfragen (German Centre of Gerontology, DZA), Berlin, Germany; ^2^Department of Sociology, Institute for East European Studies, Free University Berlin, Berlin, Germany; ^3^Department of Social Work, Protestant University of Applied Sciences Ludwigsburg, Ludwigsburg, Germany

**Keywords:** mature entrepreneurship, self-employed workers, female entrepreneurs, gender, small business

## Abstract

Mature female entrepreneurs represent a non-traditional model of self-employed workers in both ways: in terms of gender *and* age. The transition into self-employment for women aged 45 years and older represents a topic of aging research that still tends to be overlooked. Previous studies found ambivalent results for the issue regarding motives and entrepreneurial pathways between former employmen or unemployment–and starting one's own business and the ways in which these entrepreneurial activities are shaped by social differences (such as gender) and biographically accumulated resources and restrictions. This article studies biographical-related factors and motivations that determine what is variously referred to as mature entrepreneurship for men and women aged 45 and above. Using data from the German Socio-Economic Panel (SOEP), the descriptive analysis explains the main gender differences among people within the target age group who have taken the step into self-employment. The multivariate analysis interrogates the main determinants that govern any increase in the probability of becoming self-employed after the age of 45 and seeks to identify the main differences between women and men in relation to such determinants. The results show substantial gender-based occupational segregation in entrepreneurship patterns in this age group, with men working longer hours on average than women and enjoying higher average earnings. However, the multivariate analysis shows that the main drivers for mature entrepreneurship are similar for both men and women and that necessity represents an important factor for everyone for starting a business.

## Introduction

Against a background of increasing discontinuity in employment biographies and in a world of social policy instruments to delay retirement, taking the step into entrepreneurship is becoming a “new normal” in aging research on labor participation at older ages (Franke, 2012; Kautonen et al., 2017; Damman and van Solinge, 2018, 2019; Stypińska et al., 2019; Saiz-Álvarez and Coduras-Martínez, 2020; Maritz et al., 2021). Mature entrepreneurship, while remaining a relatively rare phenomenon, is currently attracting increasing attention from policymakers worldwide [Global Entrepreneurship Monitor (GEM), 2017, 2020, 2021].

While more and more studies have been found in recent years that look at the specific parameters and patterns of mature entrepreneurship, only limited research activities have revealed the interplay of age and gender for mature entrepreneurs (Damman and van Solinge, 2018; Meliou and Mallett, 2019, 2022; Maritz et al., 2021). Both aspects of gender and age share the label of “missing entrepreneurs,” who are understood to be groups of particularly vulnerable entrepreneurs facing many hurdles (including youth, migrants and disabled persons) (OECD and European Commission, 2021).

Current research has found ambivalent results for mature female entrepreneurship characteristics. There is still a lack of further empirical investigation into the significant gradients of gender in mature entrepreneurial activities and the ways in which gendered life courses and labor market segregation have an impact on senior motives and performance in self-employed work (Damman and van Solinge, 2018; Meliou and Mallett, 2019, 2022; Tonoyan et al., 2020; Maritz et al., 2021). Using a life course perspective, this study aims to explore the factors impacting the lower proportion of mature female entrepreneurs and find empirical evidence of the possible structural exclusion, such as “double jeopardy” (Wilks and Neto, 2013)–exclusion based on two factors: age and gender. This article therefore takes an intersectional perspective on age and gender to identify the divergences detectible in the traits exhibited by mature female entrepreneurs as compared to those of their male counterparts in Germany–as an example within the context of the Global Entrepreneurship Monitor (GEM) of a type of country with high population aging and low mature entrepreneurial activity (similarly to France, Spain, Sweden and the UK) (Saiz-Álvarez and Coduras-Martínez, 2020) and untapped potential for female entrepreneurship [Global Entrepreneurship Monitor (GEM), 2020, 2021; OECD, 2020; OECD and European Commission, 2021; KfW Bankengruppe, 2022].

Drawing on the theoretical approach in life course theory of accumulated advantages/disadvantages by Dannefer (2003), the authors examine gender differences in the biographically shaped motivations and former labor market experiences as well as the types of entrepreneurial activities such as branches or full-time/part-time start-ups. The analysis includes persons aged 45 years and above in relation to other literature dealing with the concept of mature entrepreneurs [Global Entrepreneurship Monitor (GEM), 2017, 2022; Fernández Sánchez, 2018; KfW Bankengruppe, 2022]. In the choice of our terminology, we followed the mainstream research in the area of entrepreneurship, as well as various policy documents, which often refers to the group of 45/50 plus entrepreneurs as “mature,” “older” or “senior” or even “gray” (Weber and Schaper, 2004; Global Entrepreneurship Monitor (GEM), 2017; Soto-Simeone and Kautonen, 2020). The Global Entrepreneurship Monitor from 2017 distinguishes between four age groups: 18–29 years = young, 30–49 years = mid aged, 50–64 years = seniors and 65–80 years older. In the latest Global Entrepreneurship Monitor (GEM) (2022) older entrepreneurs are defined as persons between 35 and 64 years.

Using multivariate analysis, the authors consider the determinants for transition to mature entrepreneurship with the aim of discovering whether there are any gender-related differences in the drivers behind starting up one's own business.

## Conceptual framework

This section describes two powerful conditions for our hypothesis regarding different pathways and outcomes for men and women in mature entrepreneurship–the biographies and external structures, such as gender-specific labor market segmentation, operating on the individual and macro level.

### Cumulative advantages and disadvantages over the life course

The theory of cumulative advantages and disadvantages (CAD), first formulated by Merton (1968) to explain the disparities in scientific careers in later life, has since been used extensively to explain the emergence of inequalities in outcomes stemming from accumulation of certain resources throughout the life course. Dannefer explains that cumulative advantage and/or disadvantage can be “defined as the systemic tendency for interindividual divergence in a given characteristic (e.g., money, health, or status) with the passage of time” (Dannefer, 2003, p. 327). Crystal and Shea used the term “cumulative advantage and cumulative disadvantage” to describe processes by which the effects of early economic, educational and other advantages can cumulate over the life course and lead to the creation of the “two worlds of aging” phenomenon (Crystal et al., 2017). The theoretical concept of cumulative advantage used extensively to explore the long-term effects of socio-economic status on health, income, family status or other outcome could further be useful in studying the second career transitions to self-employment and their determinants stemming from previous life stages and conditions of individuals. Patterns of inequality within and among cohorts emerge over time as products of the interplay between institutional arrangements and individual life trajectories (O'Rand, 1996). The CAD perspective opens up numerous new possibilities as regards the question of empirical and theoretical research gaps in mature entrepreneurship and could become an integral part of understanding the dynamic processes that underlie the pathways into self-employment. Research into mature entrepreneurship often emphasizes the role of accumulation of different resources over the life course, which facilitates or inhibits transition into self-employment (Franke, 2012; Tervo, 2014; Kautonen et al., 2017; Maritz et al., 2021). In this paper, the authors are proposing to utilize the perspective of cumulative advantages and disadvantages to illuminate the role of previous job patterns for men and women as the determinants for becoming self-employed at a mature age.

### Gender segregation in the labor market

Occupational gender segregation can include both horizontal segregation (similar skill level, different jobs) and vertical segregation (seniority or responsibility) (Rubery and Fagan, 2019). This gender segregation is also visible in the number of working hours women and men perform. For example, while the rate of part-time employment for men in Germany in 2019 was only 9.5 percent (OECD: 9.9), the equivalent figure for women was 36.3 percent (OECD: 25.1) (OECD, 2020), a difference that resulted in women having much shorter working hours on average than men. The combination of these two factors–occupational segregation and the higher rate of part-time working among women–results in a large gender pay gap (European Commission, 2021a,b). According to the latest available data from Eurostat, the unadjusted gender pay gap for EU27 in 2021 was 14.1 percent–a modest improvement compared to 15.8 percent in 2010 (European Commission, 2021a). In Germany, this figure remained relatively stable at 19 percent and is one of the highest among EU countries. Gender inequalities in the labor market, especially regarding the number of years in employment, work intensity and remuneration, result in the gender pension gap, the size of which also depends on the design of the pension system. Although the gender gap in pensions has narrowed gradually over the last decade, it nevertheless remains wide. According to Eurostat (2020), in Germany, women receive on average 37 percent less pension income than men.

This occupational and sectoral segregation by gender has exhibited surprising persistence over time and is thought to produce suboptimal development and use of skills for both men and women (European Commission, 2021b). However, segregation may also provide a degree of protection for some women's employment during recessions or times of transition, as these have an uneven impact on different sectors and occupations.

By using a life course approach to explore different pathways into mature self-employment for women and men, we argue that life course experiences and former stages in working life must be considered differently for female and male mature entrepreneurs. This combination of both aspects–life course and gender perspectives–underlines the biographical and normative context of entrepreneurship.

## Gender aspects in mature entrepreneurship in the literature

The likelihood of becoming an entrepreneur in mature ages depends on a variety of socio-demographic, professional and personal characteristics and on a range of economic, health and structural factors (Fuchs, 1982; Zhang, 2008; Wainwright and Kibler, 2014; Global Entrepreneurship Monitor (GEM), 2017; Halvorsen and Morrow-Howell, 2017; Kautonen et al., 2017; Soto-Simeone and Kautonen, 2020). This can be especially assumed for women with their interrupted working biographies (Damman and van Solinge, 2018). Starting with the desire to become self-employed in later life, entrepreneurship research in general focuses on two patterns of motivation, namely opportunity entrepreneurs and pull factors or necessity entrepreneurs and push factors (Ahmad et al., 2014; Harms et al., 2014; Moulton and Scott, 2016; Soto-Simeone and Kautonen, 2020; OECD and European Commission, 2021). Both patterns can be linked to former (working) life experiences. While “necessity” or “pushed” entrepreneurs initially start a business due to a lack of an alternative income source, former unemployment, age discrimination in the labor market or the risk of losing their jobs, “opportunity” or “pulled” entrepreneurs are defined as individuals coming from former employment or from university/college who are seeking promising career opportunities (Global Entrepreneurship Monitor (GEM), 2017). In general, studies show that opportunity entrepreneurs are more privileged in terms of financial and human capital, as well as psychological resources, whereas necessity entrepreneurs are typically less privileged in those areas and more exposed to adverse environmental push factors, such as involuntary job loss or early retirement and racial, gender or age discrimination (Damman and van Solinge, 2018; Gimmon et al., 2018). With regard to the current literature on the motivation of mature entrepreneurs, studies found evidence for both push and pull factors (Singh and DeNoble, 2003; Franke, 2012; Kautonen et al., 2017; Damman and van Solinge, 2018, 2019; Soto-Simeone and Kautonen, 2020). For example, European data underline that motives for female and mature entrepreneurs are more necessity-driven, while other studies underline non-monetary motives for older individuals, such as autonomy, usefulness or emancipation (Damman and van Solinge, 2019; Soto-Simeone and Kautonen, 2020; Meliou and Mallett, 2022). However, deeper empirical evidence regarding the proportions of mature female entrepreneurs involved in necessity or opportunity entrepreneurship remains untapped.

More information is provided regarding typical characteristics of late female entrepreneurship. Women's businesses seem to concentrate on low-wage and low-growth industries, particularly in the service sector, with women often choosing to work in health, culture and social services segments (Sappleton, 2009; Damman and van Solinge, 2018; OECD and European Commission, 2021). In addition, women are more likely than men to set up small businesses on their own as sole traders and/or as part-time entrepreneurs (Curran and Blackburn, 2001; Franke, 2012; KfW Bankengruppe, 2022).

Related to these findings, research argues that the tendency for women in regular employment to enroll in “gender-typical occupations” is also true for self-employment (Stomeyer, 2007; Kautonen et al., 2017; Damman and van Solinge, 2018) and that technology and the data-driven fourth industrial revolution will give rise to new manifestations of gender segregation in the very near future (Rubery and Fagan, 2019). It can thus be assumed that the patterns of segregation found in employment are mirrored in self-employment, with the types of businesses launched by men and women reflecting the pre-existing sectoral and occupational segregation of the labor market (Tonoyan et al., 2020).

One study shows that women tend to enter non-managerial and non-professional (gender-typical) self-employment to balance work and family demands, but that this tendency is not supported in the groups of women who are single and/or childless (Budig, 2006). Other studies suggest that gender-based labor market segregation is likely to shape beliefs about how easy it would be to start a business, and that such beliefs held by men will be more favorable, on average, than those held by women (Tonoyan et al., 2020; Maritz et al., 2021). Thus, current research has demonstrated that, as the result of gender-based labor market segregation, women are less likely than men to accumulate entrepreneurship-relevant resources, experience entrepreneurial career, or be exposed to entrepreneurial opportunities (ibidem). Even if some studies underline the emancipatory character of self-employment transitions for older women (Meliou and Mallett, 2022), women face double discrimination in terms of both age and gender and thus perceive the ease of their start-up less favorably than men (Maritz et al., 2021).

When focusing on the accumulated resources for mature entrepreneurship, *chronological age* itself and the percentage of mature persons as a proportion of the population have both been shown to be factors that influence the odds in favor and the expected benefit of starting one's own company and that are not yet fully understood in terms of differences between the genders (Lévesque and Minniti, 2006). Lévesque and Minniti (2006) introduced a theoretical model for *risk aversion* over one's life course, revealing that the likelihood of becoming an entrepreneur decreases with age (see also Maritz et al., 2021). They argue that, since everyone's lifetime is finite, the opportunity cost of time increases as one gets older. For mature female entrepreneurs, it can be assumed that individuals experiencing particular “push” pressures seem more likely to perceive themselves as being too old to start a business (Kautonen, 2008, 2013), but also indicate hopes of a second career and of achieving more autonomy (Meliou and Mallett, 2022).

*Educational attainment* has a significantly stronger effect in itself on mature entrepreneurs as compared to the general population, to the extent that one could even interpret the education factor as a form of “encouragement” to the would-be entrepreneur to start a business (Cerveny et al., 2016). According to the commonly defended argument, former homemakers and women with interruptions in their working biographies are particularly likely to suffer from a lack of up-to-date skills and qualifications when starting a business (Maritz et al., 2021). Consequently, these women will tend to suffer from lower levels of self-confidence, a lack of experience and insufficient qualifications compared to male entrepreneurs coming out of employment in a leadership position, with the goal of self-realization and the desire for professional independence very much in their minds (Franke, 2012; Meliou and Mallett, 2022). At the same time, the study by Buttler and Sierminska (2019) points out that flexibility, a job matching their competences and opportunities for growth are more important motives for female entrepreneurs, while their male counterparts are seeking independence, more income and less stress in self-employed work.

*Higher income*, access to capital or, conversely, debt seem to have a significant effect on transitions into self-employment for both mature men and women (Zissimopoulos and Karoly, 2007; Global Entrepreneurship Monitor (GEM), 2017; Buttler and Sierminska, 2019; KfW Bankengruppe, 2022). Due to interruptions in their working careers (involving high levels of unpaid and/or part-time work), a high number of women are less able to accumulate savings to use later for their own businesses (Franke, 2012; Buttler and Sierminska, 2019). In addition, problems in obtaining credit (Franke, 2012; Buttler and Sierminska, 2019), ageism and age discrimination are posing general significant barriers to entry into mature entrepreneurship (Kautonen et al., 2014; Maritz et al., 2021; Meliou and Mallett, 2022).

One crucial factor in determining a switch to self-employment is having previous experience of running one's own company at some earlier stage of life (“*serial entrepreneurship*”). It is clear then that past experience in self-employment (also in terms of simultaneous small-scale businesses) has a significant and positive impact for both men and women on the probability of becoming self-employed (Tervo, 2014; Buttler and Sierminska, 2019). According to Shaw and Sørensen (2019), women benefit especially from having previous experience in self-employment as compared to their less experienced female peers. Other major advantages connected with the prior experience of mature entrepreneurs include their better rounded *social networks* and more highly developed *technical and managerial skills* (Weber and Schaper, 2004; Kautonen, 2008; Ahmad et al., 2014; Harms et al., 2014; Halvorsen and Morrow-Howell, 2017). However, a lack of up-to-date knowledge of the business sector, problems relating to their capacity to acquire customers, or a lack of appropriate skills can all act as obstacles to business creation (Kautonen, 2008; Franke, 2012; Maritz et al., 2021).

*Earlier career trajectories* also exert an influence through their power to explain gender differences in types of entrepreneurial activity undertaken, especially in terms of the social and human capital that a new entrepreneur may or may not have had the opportunity to accumulate at his or her previous workplace (Kautonen et al., 2017; Meliou and Mallett, 2022). The type of work experience that male or female entrepreneurs gain in the time leading up to starting a business may have important implications for the nature of the venture they ultimately establish. “Since most business owners start up in their 30 or 40 s, the workplace can be seen as a formative setting where entrepreneurs accrue knowledge and contacts upon which they can draw when starting their own firms” (Sappleton, 2009, p. 194). Research by Logan (2014) in the UK demonstrates that previous careers can also pose a considerable barrier to setting up one's own company at later stage. Logan's study showed that women who had previously worked in public sector occupations were significantly disadvantaged in this regard, as such positions do not usually involve a great deal of employee autonomy or self-management, both of which factors turn out to be useful further down the road in establishing one's own business at the age of 50 or later. This observation of course suggests that one may expect gender differences to emerge as a result of differences between the genders in their last job before entering self-employment (see also Damman and van Solinge, 2018; Meliou and Mallett, 2022).

Interestingly, the study by Buttler and Sierminska (2019) found evidence that women in the transition to self-employed work are more likely to be single with savings. According to other studies, *being married* or *in a partnership* can make it easier for the entrepreneur to take risks–usually explained by the economic security that such a partnership often provides–and supplies other types of non-pecuniary support on which the new entrepreneur can often lean during the early stage of self-employment (Zissimopoulos and Karoly, 2007). The financial situation of the potential entrepreneur's family has a crucial influence on potential businesswomen, as some female entrepreneurs are only in a position to start up their business with the support of the buffer provided by their respective partners. Equally, however, single women in particular can often find themselves confronted by a precarious income situation that can end up forcing them into self-employment (Franke, 2012). Yet *family obligations* play an ambivalent role in female entrepreneurial activities (Buttler and Sierminska, 2019). Having children under 18 years of age living in the same household seems to act as a barrier to taking the risky step into self-employment, though some mature entrepreneurs may feel their working life has the happy effect of freeing them to some extent from family stresses (Bruce et al., 2000; Franke, 2012; Meliou and Mallett, 2022). Aside from that, it seems that mature female entrepreneurs are often motivated by a variety of critical events in their family lives, including divorce or “empty-nest” syndrome (Franke, 2012). This suggests that many women only begin to consider the option of starting up their own business once they have been freed of the burden of childcare responsibilities. On the other hand, self-employment also seems to provide more flexibility, helping to reconcile work and family obligations and providing more freedom to adapt working arrangements to suit one's other needs (Fernández Sánchez, 2018; Meliou and Mallett, 2022; KfW Bankengruppe, 2022).

## Empirical analysis

### Hypothesis

The literature shows that gender differences exist in the type of entrepreneurial activities that mature entrepreneurs tend to undertake, as well as in the factors that determine their transition into self-employment. Moreover, the occupational segregation that was a factor earlier in one's career may have a significant impact on the types of business that men and women decide to establish when 45 years or older. This earlier occupational segregation produces the effect that men and women accumulate their work experience in different sectors and that the businesses they end up starting in later life will naturally be conditioned by those differing experiences. We therefore expect to observe similar gender-based occupational segregation among mature entrepreneurs in Germany (*hypothesis* 1) in the analysis. In addition to this, since women are more likely to have done part-time work in their younger years, they may also be expected to be more likely to become part-time workers in self-employment in later life, too (*hypothesis 2*). These two phenomena (i.e., occupational segregation and shorter working hours) lead on to a third hypothesis, i.e., that self-employed men will on average earn more than their female counterparts (*hypothesis 3*). The wide range of research results described earlier provide inspiration for yet another hypothesis: that the drivers that lead one to become a mature entrepreneur will tend to be different for women than they are for men (*hypothesis 4*). Given the fact that women tend to start out from a less advantageous position, with family obligations and higher risk aversion, it may be expected that they will tend to be necessity-driven entrepreneurs to a greater extent than men (in view of their less extensive work experience and lower income and educational attainment, as well as their less advanced management skills).

### Data and method

The data used for the analysis are taken from the German Socio-Economic Panel (SOEP) data set for the 1984–2016 period. The SOEP provides representative longitudinal information on private households in Germany (Goebel et al., 2018). Individual information on labor status is collected on a yearly basis, meaning that changes in labor status (including switches to self-employment) can be traced.

Given the research question at issue, it makes sense to take advantage of the SOEP's longitudinal structure. From the data set defined by all people aged 45 and older, the authors have extracted the subset of those among them who have become entrepreneurs, retaining the rest of the sample as a control group. Only those individuals with no experience of self-employment within the last two years prior to their entry into self-employment are included in the set of entrepreneurs. For our purposes, entrepreneurs are defined as people who have become self-employed–whether or not they have taken on employees–according to the data on their current occupational status. Family members assisting such entrepreneurs are not considered self-employed. Personal information on individuals is provided in the data on their past employment history, along with their earnings and the positions that each person held in previous employment before the age of 45. The sample is restricted to individuals for whom information is available on their past employment. This stipulation imposes a restrictive selection criterion on the sample, as only individuals with past employment experience are included as a result.

The sample consists of 3,884 men and 3,604 women aged 45 and older. Within this sample, a total of 218 men and 185 women became self-employed between the ages of 45 and 74. However, information on occupation and number of working hours is not available for all self-employed men and women.

The variables used for the empirical analysis (both descriptive and multivariate) are listed in Table A1. Some of the variables (such as occupation, earnings and working hours while in self-employment, socio-economic status, labor market experience, past self-employment experience and propensity to accept risk) are measured at the time the individual became self-employed. Other variables (such as employment status and health satisfaction) are measured for the year before their transition into self-employment. Yet another group of variables is measured for the person's last job before going into self-employment (i.e., whether the person was a manager, along with metrics on their job satisfaction and earnings in their last job).

The descriptive analysis aims to identify the sectors in which mature entrepreneurs tend to be active and to assess whether there are any gender differences in the types of activity they undertake. Additionally, we compare income and working hours in self-employment against wages and working hours in the entrepreneurs' last jobs before entering self-employment. The results are weighted using cross-sectional SOEP weightings in order to correct for differing design probabilities in the various SOEP subsamples.

The regression estimations reveal the determining factors in becoming self-employed from the age of 45 onwards, as well as showing whether there are any detectible gender differences in such factors. The data set is used in repeated cross-sections, considering only the first transition into self-employment in cases where there were several such transitions in a person's later life course. This ensures that only one transition into self-employment is included per person. This analysis uses separate probit models with robust standard errors for men and women and a fully interacted model in which gender is interacted with all covariates. As far as the explanatory variables are concerned, in addition to considering socio-economic determinants such as age, education, partnership status, health and household income, we also control for employment situation before beginning self-employment and the major features of the person's last job, as well as each individual's willingness to take on risk.

### Empirical results

Table 1 shows a classification of the occupations in the sample, which underlines that both male and female self-employed people very often moved into their entrepreneurial activities from managerial and professional occupations (legislators, senior officials and managers, professionals, technicians and associate professionals). This is the case for approximately 70 percent of self-employed men and women. There are, however, differences between the occupations pursued by men as compared to women: while men were more strongly represented in technical professions (in physics, mathematics and engineering sciences), women tended to work in the health sector. Women were also more likely to work as “general managers,” a category very often used in reference to people running small businesses. There were also some gender differences among the 30 percent of the entire sample working in occupations other than managerial and professional occupations. While 14 percent of women worked in the service sector (in jobs relating to childcare, retail sales or hairdressing), only 7 percent of self-employed men worked in this sector. On the other hand, 10 percent of the self-employed men were occupied in craft and related trades (such as construction work, and as painters or electricians), while almost no self-employed women (3 percent) had held down occupations in that sector. This contrast chimes well with hypothesis 1.

**Table 1:** Occupations of older entrepreneurs (ISCO Classification) by gender.

	**Men**	**Women**
1 Managers	15.9	22.7
2 Professionals	33.8	20.4
3 Technicians and associate professionals	22.0	33.3
4 Clerical support workers	0.8	2.3
5 Service and sales workers	7.0	13.9
6 Skilled agricultural, forestry and fishery workers	5.2	1.8
7 Craft and related trades workers	9.6	2.6
8 Plant and machine operators, and assemblers	3.9	0.1
9 Elementary occupations	1.8	3.0

Source: SOEP 1984–2016, own calculations. Weighted frequencies. N = 196 (men), 159 (women). The difference between men and women in the distribution of occupations is statistically different (p < 0.000).

Table 2 shows a comparison of earnings and number of hours worked both before entering self-employment and during it. A comparison of earnings before becoming self-employed reveals large differences between the genders. While men earned an average of 2,637 euros per month before becoming self-employed, their female peers earned only 911 euros. In relation to number of working hours, men worked 45 h per week on average, while women spent only 29 h at work. When one compares current monthly earnings from self-employment against past earnings, one observes that self-employed women earned 113 euros less on average than they did before they entered self-employment yet worked almost the same number of hours. Men also faced some reduction in earnings (about 343 euros per month), but they also worked 6 h fewer per week on average once they began self-employment. As a result, the average hourly earnings of women and men remain stable after the transition. These results confirm hypotheses 2 and 3 and the biographical relationship of entrepreneurship with typical female working careers, since self-employed women, due to their higher rate of part-time entrepreneurship, worked fewer hours on average than men and therefore earned less money.

**Table 2:** Earnings and working hours of older entrepreneurs.

	**Before self-employment**							**During self-employment**						
	**Women**			**Men**			**Mean diff**.	**Women**			**Men**			**Mean diff**.
	**N**	**Mean**	**Std. dev**.	**N**	**Mean**	**Std. dev**.	***p*** **-value**	**N**	**Mean**	**Std. dev**.	**N**	**Mean**	**Std. dev**.	***p*** **-value**
Average earnings (monthly) – EUROS	184	€911	776	215	€2,637	2,758	0.000	184	€798	745	215	€2,294	2,935	0.000
Average number of working hours per week	176	29 h	15	210	45 h	13	0.000	165	28 h	19	201	39 h	21	0.000

Source: SOEP 1984–2016, own calculations. Weighted frequencies.

Next, with the aim of identifying the main factors that determine transitions into self-employment for each of the two genders, we estimated a probit model. Table A2 in the Appendix^1^ presents a comparison of the determinants included in the regression (socio-economic features, labor market experience and propensity to accept risk), both between men and women and between the self-employed and the control group.

Table 3 shows the results of the probit estimations. The dependent variable takes a value of 1 if the relevant person becomes self-employed and 0 otherwise. The first step in the analysis was to estimate the model separately for men and for women.

**Table 3:** Multivariate analysis of the determinant factors of late entrepreneurship. Probit. Women and men.

**Self-employment**	**Women**			**Men**		
	**Coef**.	**Std. Err**.	**P>|z|**	**Coef**.	**Std. Err**.	**P>|z|**
Age categories (ref. 45–54)						
2: 55–64	−0.118	0.085	0.164	−0.250	0.093	0.007
3: 65+	−0.090	0.163	0.583	0.071	0.151	0.639
West Germany	0.160	0.078	0.041	−0.006	0.067	0.924
German nationality	0.038	0.132	0.771	−0.146	0.100	0.144
Education (ref. Low income)						
2. Mittel	0.120	0.094	0.200	0.230	0.114	0.043
3. Hoch	0.352	0.109	0.001	0.371	0.129	0.004
Partner in the HH /Other	−0.125	0.076	0.099	−0.045	0.094	0.628
Log. HH income	0.137	0.066	0.037	0.364	0.071	0.000
Status last year (ref. Employed)						
Not employed	0.222	0.098	0.023	0.622	0.195	0.001
Unemployed	0.183	0.116	0.116	0.850	0.093	0.000
Others	−0.142	0.149	0.340	−0.160	0.132	0.226
Health Staisfaction in t-1	0.018	0.013	0.180	0.014	0.014	0.323
Experience in Full time	−0.001	0.003	0.752	0.005	0.005	0.347
Past self-employment experience	0.103	0.012	0.000	0.067	0.009	0.000
Risk propensity	0.062	0.013	0.000	0.071	0.013	0.000
Manager experience in last job	0.205	0.143	0.153	0.247	0.076	0.001
Wage in last Job	−0.200	0.035	0.000	−0.033	0.054	0.536
Job Satisfaction in last job	−0.041	0.012	0.001	−0.009	0.014	0.502
Cohort (ref. Born before 1956)						
Born between 1956 and 1964	0.033	0.087	0.705	−0.147	0.083	0.076
Born between 1965 and 1971	0.015	0.107	0.889	−0.024	0.104	0.815
Constant	−3.050	0.627	0.000	−6.785	0.690	0.000
*Pseudo R2*	0.080	0.104
*N*	37.346	35.566

Source: SOEP 1984–2016, own calculations. Robust standard errors.

In the case of women, *the age categories* did not have an influence on the probability of becoming self-employed. The probability of becoming self-employed was higher in West Germany than in the East of the country. However, *nationality* did not appear to have any significant effect on that probability. In a similar pattern to the results shown in Table 3, women with higher *educational attainment* and a higher *household income* showed a higher probability of becoming entrepreneurs. Against this, the presence of a partner in the household had a negative effect on that likelihood. Having a *non-employed status* increased the probability of becoming self-employed in the case of women. Furthermore, the higher the *wages* and *job satisfaction* in a woman's last job, the lower the probability of her becoming self-employed. Finally, one's *willingness to take risks* was also a significant determinant for self-employment, even after controlling for the other variables.

The results for men show that *age* closer to retirement (being between 55 and 64 years old) had a negative impact on the probability of becoming self-employed, while *high levels of education* and *household income* had a significant positive effect on the same probability. While, for women, being non-employed increased the probability of becoming self-employed, both conditions of being *non-employed* and *unemploye*d had a positive effect for men for transitioning into self-employment. It turns out that *willingness to take risk* was a determining factor in self-employment for men as for women. However, in contrast to women, it seems that having *managerial experience* increased the probability of men becoming self-employed.

*Previous experience in self-employment* had a significant and positive effect for both men and women. However, the *cohorts* had no significant effect for either gender (its impact is significant only for men born between 1956 and 1964), which suggests that the probability of becoming self-employed did not change significantly among the cohorts considered in this study.

Our analysis did not find any significant effect that points to an individual's *state of health* acting as a deterrent to making a change in one's employment status. This finding applied to both women and men.

The estimations for the models as considered separately for men and women show that the main drivers for mature entrepreneurship were similar for both genders and that necessity (in terms of non-employment and unemployment) represented an important factor in starting a business for men and women alike. These results are contrary to our hypothesis as regards the gender differences in drivers for mature entrepreneurship (hypothesis 4). In order to test differences in impact across genders statistically, we also conducted estimates on a fully interacted model as the next step in the analysis, i.e., we interacted gender with all other covariates in the model. The results of these estimations are shown in Table A3 in the Appendix.

**Figure 1:**
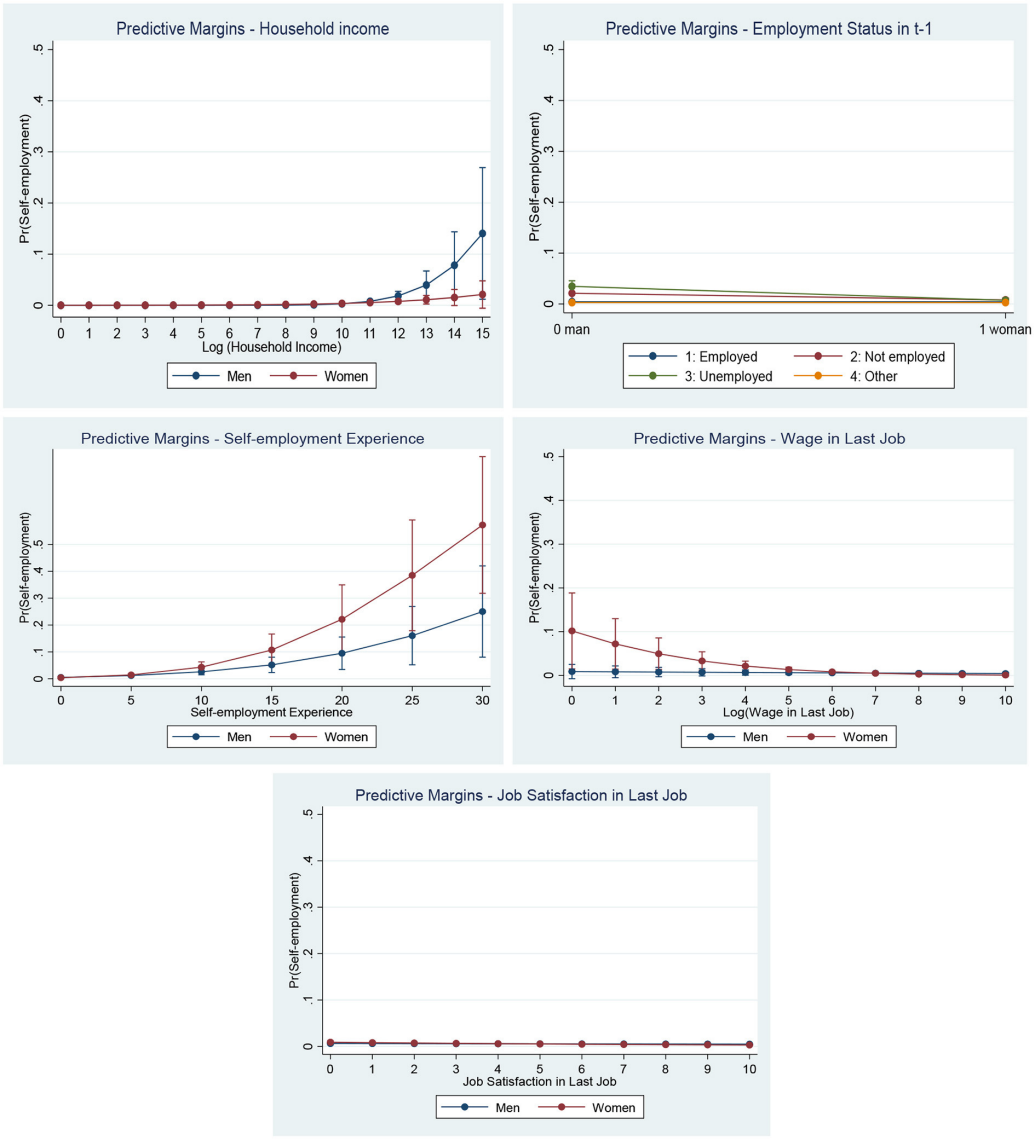
Predictive margins of the significant interaction effects (Table A3). Source: SOEP 1984–2016, own calculations. Robust standard errors.

The coefficients of the interacted terms represent the differences in the size of effects across the two genders. We found no gender differences in the interacted effects that are not significant. This observation applies to the effects of age, region, nationality, education, household structure, health satisfaction, full-time job experience, propensity to take on risk and managerial experience. However, one does find differences between men and women in relation to the effects of household income, past employment status and experience in self-employment, as well as in the effects of earnings and job satisfaction in one's last job. In order to better understand the differing gender effects on the probability of becoming self-employed, we have plotted the marginal effects of the gender differences that the analysis showed up as significant (Figure 1).

In the first graphic, one can see the predictive margins for varying values of the logarithm of household income. One observes no difference in the predictive margins between men and women at lower household incomes. However, men who enjoyed a high household income showed a higher (predicted) probability of becoming self-employed than women. Even if this impact is a weak one (that is to say that the predicted margins were small), it does constitute evidence that men enjoying a privileged financial situation are more likely to become entrepreneurs, while this is not the case for women.

In the second graphic, one observes the predicted margins for the various employment statuses in the sample at t-1. Here, too, the gender differences are small. As can be seen in Table 3, being unemployed predicted a higher probability of becoming self-employed for men (but not for women), while having non-employed status predicted a higher probability of becoming self-employed for both men and women. The latter effect was smaller for women than for men. Necessity seems to be a relevant factor for both men and women, but both non-employment and unemployment lead to higher probabilities of becoming self-employed for men than they do for women.

The largest effects on self-employment (and the largest differences between the genders) are observed in the experience factor. The greater an individual's past experience in self-employment, the higher the probability of him or her becoming self-employed. This effect was larger for women than for men.

Earnings from one's last job also had only a small effect on the probability of becoming self-employed, although here, too, there were significant gender differences. For men, the effect was close to zero, but for women one can observe that the lower the wage, the higher the probability of becoming self-employed.

Job satisfaction (in one's last job) had a significant effect for women (Table 3), but not for men. The interaction in the fully interacted model was significant. However, the effect and, to be more specific, the size of the coefficient was close to zero (Figure 1).

From the results of the fully interacted model, the authors conclude that some gender effects are observable, but are (a) small and (b) mainly differences in terms of strength rather than direction, which again goes against the fourth hypothesis.

## Discussion

The current state of research in the field of mature entrepreneurship indicates gender-specific differences in pathways and patterns of transitioning to self-employed work. Nevertheless, there is still an empirical research gap regarding the influence as regards biographically determined career paths for women and men.

Using a life course approach, we have hypothesized that biographical differences between men and women in their participation on the labor market also have implications for the entrepreneurship activities that they take on when 45 years or older.

In summary, our findings show a clear gender occupational segregation in mature entrepreneurs. This reflects the gender-segregated experience accumulated before becoming a mature entrepreneur. At the same time, it also reflects the different restrictions and opportunities that men and women face when starting a business as a second career option–found, for example, in earlier works by Damman and van Solinge (2018), Maritz et al. (2021) or Meliou and Mallett (2022).

Our results show that gender differences in working time and gender pay gap are also a reality for mature entrepreneurs. On average, men work 39 h weekly, while women do not reach 30 h on average. As a result of the occupational segregation and the differences in working time, there are also notable gender differences in earnings. The transition to mature self-employment is associated with income losses for both men and women, which means that mature entrepreneurship does not help to reduce the gender pay gap (Kautonen et al., 2017).

An important focus of our analysis is on the factors affecting the transition into entrepreneurship and how they differ between female and male mature entrepreneurs. To this end, we have analyzed the role played by the former employment status in the probability of such a transition. On that question, having a non-employed or an unemployed status increased the probability of becoming self-employed for women and men, a finding that tends to support the view that the decision to become a mature entrepreneur is motivated at least in part by necessity or by the absence of any alternative options (OECD and European Commission, 2021). These findings contrast with the results of, for example, Damman and van Solinge (2019) or Soto-Simeone and Kautonen (2020), who highlight key entrepreneurial drivers as the desire for autonomy or usefulness. This is an unexpected result, since gender differences in the employment biographies before starting one's own business and the dissimilitude in the entrepreneur activity might lead one to believe that drivers are different for men and women. At the same time, the findings may also indicate that the categories of “opportunity” and “necessity” are more complex, as we do not have further information on the internal motives in the data. For example, Damman and van Solinge (2018) underline the non-linearity in the life courses of female entrepreneurs, while Meliou and Mallett (2022) point to the fulfillment of personal goals and the emancipatory character of women stepping into self-employed work, including from a former status of unemployment. Against this background, the finding that the absence of any other person in the household increases the probability of becoming self-employed in the case of women only is an interesting one–the step into self-employment could therefore be interpreted as a solution for achieving a household income, but also as a step toward personal independence.

The results of the analysis also suggest that people employed in well-paid jobs are less likely to become entrepreneurs, thus reinforcing the idea that self-employment is something that one enters out of necessity. A negative effect on the probability of becoming self-employed when there is high job satisfaction in the former job, however, could also be interpreted, for example, in light of the study by Buttler and Sierminska (2019). Both authors found that female entrepreneurs in particular are interested in matching their skills and competences with their self-employed work and in personal fulfillment, while for men a higher income and career prospects are also decisive factors.

Another surprising result is that the authors' analysis did not find any (negative) significant effects of the individual's state of health on the probability of starting a business. However, it should be noted that only one health-related variable was included in the analysis.

The existing literature reveals that critical life events, biographical turning points and general dissatisfaction with one's current employment frequently appear as crucial and somewhat interlinked factors in whether women begin rethinking their ideas, needs and wishes in relation to possible self-employment (Franke, 2012; Meliou and Mallett, 2022). Most studies speak to the more formidable barriers faced by women in their transition into self-employment in comparison to men (Kautonen et al., 2017; Damman and van Solinge, 2018; Maritz et al., 2021). It may be argued that women aged 45 and older have usually accumulated less work experience and less practice in managerial roles, have a lower level of financial security and are usually active in lower-paid and lower-growth industries than men, and that all these factors leave them with inferior resources available for founding a new business. However, the results of the multivariate analysis show that the factors determining entry into self-employment do not differ very radically between men and women. Even if one can observe some differences between the genders in relation to such effects, those differences are small and all point in the same direction.

### Theoretical implications

Our findings contribute to the accumulation of theoretical knowledge regarding gender segregation and gender gap in relation to self-employment. This problem has often been overlooked, with the majority of the literature focusing on occupational gender segregation in employment (Stomeyer, 2007). Specifically, our aim is to contribute to a better understanding of entrepreneurial gender segregation from the life course perspective, which provides a unique insight into the way inequality and segregation determine the entrepreneurial paths for women aged 45 and older. Researchers have dedicated significant attention to comparing businesses owned by men and those owned by women on the basis of the performance and growth of their firms (Bird and Sapp, 2004; Damman and van Solinge, 2018; Sappleton, 2018). However, research has been missing on the differences between men and women with regard to antecedents of entrepreneurship and the biographical experiences relevant to establishing businesses in later life, such as previous work status or experience of unemployment, income and working hours in the last job, and the role of a partner or family as elements which influence the entrepreneurial motivations (Halvorsen and Morrow-Howell, 2017). This paper contributes to the theoretical knowledge about the way mechanisms of labor market segregation transfer into the entrepreneurial sector, resulting in persisting inequalities between genders.

First and foremost, this study revealed that gender segregation takes on several forms in the case of mature entrepreneurs, which enhances our knowledge about the way entrepreneurial activities are gendered. As hypothesized, the results of our analysis not only show occupational segregation, with women flocking to traditional female sectors, such as health care, but also confirm that the gender pay gap persists after transition to self-employment. Furthermore, we identified a gap in the number of working hours between genders, which does not translate into income, suggesting that women work more and earn less in self-employment. These findings are in line with the majority of the literature on vertical and horizontal gender segregation in the labor market (Watts, 2005; Larsen, 2006; Schäfer et al., 2011). However, they extend the empirical analysis and theoretical implications to the realm of entrepreneurship.

Secondly, our findings about the primary motivations of mature entrepreneurs can be understood in light of the life course perspective and the theory of accumulation of advantages and disadvantages (Dannefer, 2003). The results show that the “necessity” motivation for starting one's own business at an older age is similar for both women and men. This result shows that, despite various antecedents of entrepreneurial activities between men and women, the ultimate transition into self-employment after the age of 45 is motivated by unemployment or non-active status. This unexpected result indicates that the situational factors (such as being unemployed) are detrimental to the decision to become self-employed later in life and suggest that the labor market challenges for workers aged 45 and older, such as age discrimination and ageism (Harris et al., 2018), might be more prevalent in this age category than the inequalities related to gender. However, our findings also indicate possible overlapping motives driven by the simultaneity of life events, such as becoming unemployed and separation from one's partner depending if entrepreneurs are in their late forties or close to the legal retirement age. Even though our study pointed to certain differences between men and women in the way their previous experiences shape their motivation to become self-employed later in life–for example, men with managerial roles in their resumes were more likely to start a business than women, which can be viewed as confirmation of the assumptions of CAD theory–those differences were not large enough to place men in a significantly more privileged position than women.

In terms of the theoretical contribution made by this paper, we hope to have deepened the understanding of intricacies at the intersection of gender, late entrepreneurship and life course analysis by providing empirical evidence which partially confirms the hypothesis about gender segregation in the entrepreneurial context, but at the same time provides insights into the similarities in the paths to self-employment in later life between men and women. This finding confirms both that age has a stronger influence than gender on the way mature entrepreneurs experience their transition in their second careers and that the commonality of the experience of unemployment could be a decisive factor for such a transition. The experience of age discrimination in the labor market could shed some light on the fact that, contrary to what we initially assumed, men are also more likely to turn to self-employment out of necessity, which could potentially be the result of not finding suitable employment alternatives due to age and closeness to retirement. Despite the fact that most of the research on gendered ageism focuses on “double jeopardy” experiences for mature women (Riach et al., 2015; Maritz et al., 2021; Rochon et al., 2021), little attention is paid specifically to men's experiences of age discrimination on the labor market. In future research, it might be worthwhile exploring the ways in which men experience age discrimination in employment differently to women, since age discrimination might be the first time they are faced with exclusion or disadvantage in their professional lives (provided they are not exposed to racial, ethnic, religious or sexual orientation discrimination).

Last but not least, our study provides empirical illustration of the theoretical conceptualizations of mature entrepreneurship which have been introduced lately to the relevant literature. In our analysis, we deployed several categories from the model of self-employment in later life by Halvorsen and Morrow-Howell (2017) by looking at both individual antecedents (such as age, work history or risk tolerance) and contextual antecedents (e.g., family status) of entrepreneurship on the one hand and the individual outcomes of the entrepreneurial activities (such as income and working hours) on the other. Our paper responds to the claim made by Halvorsen and Morrow-Howell suggesting intense investigation of the role of previous experiences of mature adults in their path to self-employment, since those experiences are significantly different to those of employees.

### Limitations and future directions

In general, the question of opportunity and necessity entrepreneurship is therefore a very complex issue, requiring a deeper understanding of life course approaches and the heterogeneity of transitions in later life. The results presented in this paper suggest further research on the various determinates of mature entrepreneurship from a gender perspective. Our analysis illustrates advantages and disadvantages within the context of female entrepreneurs aged 45 and older including various time perceptions until retirement or amortization of a business.

Our study is limited due to the fact that we have a restricted number of observations, e.g., for different age categories. The number of transitions is too small to allow for a more comprehensive analysis of occupation segregation. A higher number of transitions into self-employment would also enable us to make more efficient estimations in the multivariate analysis. Furthermore, we have only limited information regarding entrepreneurial activities and motives depending on closeness to retirement. Measures concerning the entrepreneurial network, resources that were accumulated in a person's last job or a person's hopes regarding self-employment are not part of the questionnaire. The identification of deeper life course mechanisms with regard to mature entrepreneurship was therefore limited. Moreover, our data are limited to Germany and thus tied to the specific gendered labor market conditions there and to the general entrepreneurial culture and framework of policy on entrepreneurship.

Further investigation might be useful to focus in greater detail on the gender-specific social norms, structural conditions, the interplay between different transitions (e.g., divorce and the decision to become self-employed) and individual factors such as agency over one's own life course. It would also be worthwhile looking at the similarities between women and men on their pathways to self-employment according to different age categories. Given that mature female entrepreneurs still seem to be underrepresented and in need of more social concern, it is also important to criticize the “ideal entrepreneur” model which is sometimes still prevalent and to underline the diversity that exists in entrepreneurial activities and the biographies of the persons behind it.

### Practical implications

Analysis of the factors that make people more likely to become self-employed and of the individual characteristics of those best equipped to take this option as an opportunity to improve their employment conditions can be of practical relevance to policymakers by helping them design suitable institutional support mechanisms for mature entrepreneurship.

The analysis has shown that the push of necessity is among the most important factors that determine whether or not one sets out on the road to mature entrepreneurship. This important aspect of the topic needs be taken into consideration when drawing up labor market policy with the aim of offering instruments and programs designed to provide support for mature unemployed/not-employed people who wish to start up new businesses. Aside from that, any such support programs should also take into account a number of other important aspects of the problem, including the position of people with lower levels of educational attainment and less managerial experience, who may be expected to be less likely to become entrepreneurs and who may benefit from targeted training or coaching to encourage their entry into self-employment and to facilitate their transition into the world of business (Franke, 2012; Buttler and Sierminska, 2019). In the case of women–who, as is already clear, tend to have less experience in employment and access to less extensive work networks–it is possible that potential female entrepreneurs would also benefit from well-designed supportive measures. It would also make sense to take the existence of gender-based occupational segregation into account in any effort to design policies aimed at promoting mature self-employment as a heterogeneous group with different needs and starting positions.

## Data availability statement

This study uses data from the German Socio-Economic Panel (SOEP). Restrictions apply to the availability of these data, which were used under license for the current study. Data are however available from the SOEP Research Data Center (https://www.diw.de/en/diw_01.c.678568.en/research_data_center_soep.html). The code used during the current study is available from the corresponding author on reasonable request for all interested researchers.

## Author contributions

LRG is the leading author of the paper and prepared outline of the empirical part. AF, LRG, and JS prepared the outline of the publication and introduction. AF and JS prepared outline of the theoretical part and policy background. All authors shared the preparation of discussion, conclusion, made comments, suggestions, and corrections to the rest of the article. All authors contributed to the article and approved the submitted version.

## Footnotes

^1^The number of observations for the control group (i.e., for those in the sample who did not transition into self-employment) does not correspond to the number of persons in the sample due to the fact that the data have been treated in repeated cross-sections.
