# Supplementary material for: Gender differences in the determinants of mature entrepreneurship? The case of Germany
Source: Front Sociol. 2022 Dec 7;7:998230. doi: 10.3389/fsoc.2022.998230 (PMC9768446; doi:10.3389/fsoc.2022.998230)
Supplement: Supplementary file 1 [file Data_Sheet_1.docx]

Table A1. List of variables used in the empirical analysis

| **Descriptive Analysis** | |
| --- | --- |
| **Occupation (ISCO-88)** | International Standard Classification of Occupations (ISCO) |
| **Average number of working hours** | Actual work time per week |
| **Average earnings** | Net Labour income in Euro - Monthly |
| **Multivariate Analysis** | |
| **Age** | Age categories 45-54; 55-64; 65-74 |
| **Cohorts** | Born before 1956; born between 1956 and 1964; born after 1964 |
| **West Germany** | Person lives in West Germany (=1); Person lives in East Germany (=0) |
| **German nationality** | Person has German nationality (=1); otherwise (=0) |
| **Education** | Low (=0): No Vocation Degree; Medium (=1) Vocational degree (Apprenticeship; Vocational School; Health Care School; Technical School; Civil Service Training); High (=2) University Degree (Technical College; University; State doctorate; Dual Studies) |
| **Partner in the Household** | Married with or without children and other combinations (=1); Living alnone or single parent (=0) |
| **Household income** | Household post-government income. In the regression it has been logarithmised. |
| **Employment status in t-1** | Employment status in the year before becoming self-employed. Employed (=1); Non employed (=2); Unemployed (=3) and others (like pensioners) (=4) |
| **Health satisfaction in t-1** | Health satisfaction in the year before becoming self-employed. Scale 0-Low to 10-High. |
| **Labour market experience (Full time)** | Number of years in full time employment before becomming self-employed. |
| **Past self-employment experience** | Cummulated number of years in self-employment |
| **Risk propensity** | Question for measuring risk propensity: Are you generally a person who is willing to take risks or do you try to avoid taking risks? The answer scale goes from 0 to 10 where 0 means "not at all willing to take risks" and the value 10 means "very willing to take risks". |
| **Manager in the last job** | Manager experience in last job before becoming self-employed. Scale 0-Low to 10-High. |
| **Wage in the last job** | Earnings in last job before becoming self-employed |
| **Job satisfaction in the last job** | Satisfaction with the last job before becoming self-employed. Scale 0-Low to 10-High. |
