# Supplementary material for: Gender differences in the determinants of mature entrepreneurship? The case of Germany
Source: Front Sociol. 2022 Dec 7;7:998230. doi: 10.3389/fsoc.2022.998230 (PMC9768446; doi:10.3389/fsoc.2022.998230)
Supplement: Supplementary file 2 [file Data_Sheet_2.docx]

Table A.2: Socio-economic characteristics of entrepreneurs and non-entrepreneurs (Men/Women)

|  | **Women** | | | **Men** | | | **Mean difference (p-value)** |
| --- | --- | --- | --- | --- | --- | --- | --- |
|  | Transition into self-employment | | | | | |  |
|  | No. | Mean | Std.dev. | No. | Mean | Std.dev. |  |
| Age | 185 | 54,3 | 7,47 | 218 | 56,2 | 7,9 | 0,010 |
| Age category 1: 45-54 | 115 | 61% |  | 114 | 48% |  | 0,015 |
| Age category 2: 55-64 | 49 | 23% |  | 56 | 32% |  | 0,015 |
| Age category 3: 65+ | 21 | 15% |  | 48 | 20% |  | 0,015 |
| West Germany (ja/nein) | 185 | 87% |  | 218 | 76% |  | 0,277 |
| German Nationality (ja/nein) | 184 | 96% |  | 218 | 93% |  | 0,382 |
| Education – Low | 17 | 9% |  | 9 | 5% |  | 0,007 |
| Education - Medium | 107 | 62% |  | 107 | 58% |  | 0,007 |
| Education - High | 60 | 28% |  | 100 | 37% |  | 0,007 |
| Partner in the Household (ja/nein) | 185 | 68% |  | 218 | 88% |  | 0,000 |
| Household income in t-1 | 184 | 40.608 € | 29.973 | 215 | 53.918 € | 54.258 | 0,002 |
| Employee in t-1 | 127 | 70% |  | 137 | 62% |  | 0,000 |
| Non-Employment in t-1 | 24 | 10% |  | 7 | 4% |  | 0,000 |
| Unemployed in t-1 | 15 | 5% |  | 41 | 19% |  | 0,000 |
| Other employment status in t-1 | 19 | 15% |  | 33 | 15% |  | 0,000 |
| Health Satisfaction in t-1 | 185 | 6,5 | 2,1 | 217 | 6,8 | 2,2 | 0,804 |
| Labour Market Experience (Full-time) | 184 | 15,4 | 9,9 | 218 | 30,9 | 8,8 | 0,000 |
| Risk propensity | 185 | 5,2 | 2,3 | 218 | 5,7 | 2,1 | 0,000 |
| Manager in the last job (ja/nein) | 185 | 8% |  | 218 | 15% |  | 0,000 |
| Wage in the last job | 185 | 960 € | 776 | 218 | 2.593 € | 2.758 | 0,000 |
| Job satisfaction in the last job | 185 | 6,4 | 2,3 | 218 | 7,1 | 2,5 | 0,036 |
|  | Women | | | Men | | | Mean difference (p-value) |
|  | No transition into self-employment | | | | | |  |
|  | No. | Mean | Std.dev. | No. | Mean | Std.dev. |  |
| Age | 38033 | 56,5 | 8,0 | 36123 | 56,3 | 8,0 | 0,000 |
| Age category 1: 45-54 | 19387 | 47% |  | 17135 | 47% |  | 0,000 |
| Age category 2: 55-64 | 12329 | 34% |  | 11617 | 33% |  | 0,000 |
| Age category 3: 65+ | 6317 | 19% |  | 7371 | 19% |  | 0,000 |
| West Germany (ja/nein) | 38033 | 80% |  | 36123 | 81% |  | 0,000 |
| German Nationality (ja/nein) | 38033 | 91% |  | 36123 | 91% |  | 0,000 |
| Education – Low | 5311 | 16% |  | 3254 | 11% |  | 0,000 |
| Education - Medium | 23554 | 66% |  | 22235 | 67% |  | 0,000 |
| Education - High | 8870 | 18% |  | 10442 | 22% |  | 0,000 |
| Partner in the Household (ja/nein) | 38033 | 69% |  | 36123 | 77% |  | 0,000 |
| Household income in t-1 | 38032 | 36.237 € | 24.100 | 36123 | 38.876 € | 23.431 | 0,000 |
| Employee in t-1 | 26624 | 68% |  | 25983 | 72% |  | 0,000 |
| Non-Employment in t-1 | 2103 | 5% |  | 284 | 1% |  | 0,000 |
| Unemployed in t-1 | 1885 | 5% |  | 1851 | 6% |  | 0,000 |
| Other employment status in t-1 | 7421 | 21% |  | 8005 | 21% |  | 0,000 |
| Health Satisfaction in t-1 | 37526 | 6,3 | 2,2 | 35622 | 6,3 | 2,1 | 0,881 |
| Labour Market Experience (Full-time) | 37964 | 18,7 | 12,2 | 36025 | 30,9 | 8,3 | 0,000 |
| Risk propensity | 38033 | 4,0 | 2,3 | 36123 | 4,7 | 2,2 | 0,000 |
| Manager in the last job (ja/nein) | 38033 | 2% |  | 36123 | 5% |  | 0,000 |
| Wage in the last job | 38033 | 1.197 € | 913 | 36123 | 2.165 € | 1.371 | 0,000 |
| Job satisfaction in the last job | 38033 | 6,6 | 2,2 | 36123 | 6,6 | 2,2 | 0,001 |

Source: SOEP 1984-2016, own calculations. Weighted frequencies, unweighted number of observations.
