# Supplementary material for: Gender differences in the determinants of mature entrepreneurship? The case of Germany
Source: Front Sociol. 2022 Dec 7;7:998230. doi: 10.3389/fsoc.2022.998230 (PMC9768446; doi:10.3389/fsoc.2022.998230)
Supplement: Supplementary file 3 [file Data_Sheet_3.docx]

Table A.3. Multivariate analysis of the determinant factors of late entrepreneurship. Probit. Women and men. Fully interacted model.

| **Self-employment** | **Women and Men** | | |
| --- | --- | --- | --- |
|  | Coef. | Std. Err. | P>\|z\| |
| Age categories (ref. 45-54) |  |  |  |
| 2: 55-64 | -0.250 | 0.093 | 0.007 |
| 3: 65+ | 0.071 | 0.151 | 0.638 |
| Gender (=1 woman) | 3.736 | 0.932 | 0.000 |
| Age categories (ref. 45-54)*Gender (=1 woman) |  |  |  |
| 2: 55-64*woman | 0.132 | 0.126 | 0.296 |
| 3: 65+*woman | -0.160 | 0.222 | 0.471 |
| West Germany | -0.006 | 0.067 | 0.924 |
| West Germany*Gender (=1 woman) | 0.166 | 0.103 | 0.105 |
| German nationality | -0.146 | 0.100 | 0.144 |
| German nationality*Gender (=1 woman) | 0.185 | 0.166 | 0.265 |
| Education (ref. Low income) |  |  |  |
| 2. Mittel | 0.230 | 0.114 | 0.043 |
| 3. Hoch | 0.371 | 0.129 | 0.004 |
| Education (ref. Low income)*Gender (=1 woman) |  |  |  |
| 2. Mittel*Gender (=1 woman) | -0.110 | 0.147 | 0.454 |
| 3. Hoch* Gender (=1 woman) | -0.019 | 0.169 | 0.909 |
| Partner in the HH /Other | -0.045 | 0.094 | 0.628 |
| Partner in the HH /Other*Gender (=1 woman) | -0.080 | 0.120 | 0.508 |
| Log. HH income | 0.364 | 0.071 | 0.000 |
| Log. HH income*Gender (=1 woman) | -0.226 | 0.097 | 0.020 |
| Status last year (ref. Employed) |  |  |  |
| Not employed | 0.622 | 0.195 | 0.001 |
| Unemployed | 0.850 | 0.093 | 0.000 |
| Others | -0.160 | 0.132 | 0.226 |
| Status last year (ref. Employed)*Gender (=1 woman) |  |  |  |
| Not employed*Gender (=1 woman) | -0.400 | 0.218 | 0.067 |
| Unemployed*Gender (=1 woman) | -0.667 | 0.149 | 0.000 |
| Others*Gender (=1 woman) | 0.018 | 0.199 | 0.928 |
| Health Staisfaction in t-1 | 0.014 | 0.014 | 0.323 |
| Health Staisfaction in t-1*Gender (=1 woman) | 0.004 | 0.019 | 0.846 |
| Experience in Full time | 0.005 | 0.005 | 0.347 |
| Experience in Full time*Gender (=1 woman) | -0.006 | 0.006 | 0.327 |
| Past self-employment experience | 0.067 | 0.009 | 0.000 |
| Past self-employment experience*Gender (=1 woman) | 0.035 | 0.015 | 0.017 |
| Risk propensity | 0.071 | 0.013 | 0.000 |
| Risk propensity*Gender (=1 woman) | -0.008 | 0.018 | 0.659 |
| Manager experience in last job | 0.247 | 0.076 | 0.001 |
| Manager experience in last job*Gender (=1 woman) | -0.042 | 0.162 | 0.795 |
| Wage in last Job | -0.033 | 0.054 | 0.536 |
| Wage in last Job*Gender (=1 woman) | -0.167 | 0.064 | 0.009 |
| Job Satisfaction in last job | -0.009 | 0.014 | 0.502 |
| Job Satisfaction in last job*Gender (=1 woman) | -0.031 | 0.018 | 0.088 |
| Cohort (ref. Born before 1956) |  |  |  |
| Born between 1956 and 1964 | -0.147 | 0.083 | 0.076 |
| Born betwenn 1965 and 1971 | -0.024 | 0.104 | 0.815 |
| Cohort (ref. Born before 1956)*Gender (=1 woman) |  |  |  |
| Born between 1956 and 1964*woman | 0.180 | 0.120 | 0.134 |
| Born betwenn 1965 and 1971*woman | 0.039 | 0.150 | 0.793 |
| Constant | -6.785 | 0.690 | 0.000 |
| *Pseudo R2* | 0,091 | | |
| *N* | 72.912 | | |

Source: SOEP 1984-2016, own calculations. Robust standard errors.
